# Classification of Electrophysiological Signatures With Explainable Artificial Intelligence: The Case of Alarm Detection in Flight Simulator

**DOI:** 10.3389/fninf.2022.904301

**Published:** 2022-06-16

**Authors:** Eva Massé, Olivier Bartheye, Ludovic Fabre

**Affiliations:** Centre de Recherche de l’Ecole de l’Air, Salon-de-Provence, France

**Keywords:** single-trial classification, pBCI, inattentional deafness, brain activity, ERP, explainable AI

## Abstract

Relevant sounds such as alarms are sometimes involuntarily ignored, a phenomenon called inattentional deafness. This phenomenon occurs under specific conditions including high workload (i.e., multitasking) and/or cognitive fatigue. In the context of aviation, such an error can have drastic consequences on flight safety. This study uses an oddball paradigm in which participants had to detect rare sounds in an ecological context of simulated flight. Cognitive fatigue and cognitive load were manipulated to trigger inattentional deafness, and brain activity was recorded *via* electroencephalography (EEG). Our results showed that alarm omission and alarm detection can be classified based on time-frequency analysis of brain activity. We reached a maximum accuracy of 76.4% when the algorithm was trained on all participants and a maximum of 90.5%, on one participant, when the algorithm was trained individually. This method can benefit from explainable artificial intelligence to develop efficient and understandable passive brain–computer interfaces, improve flight safety by detecting such attentional failures in real time, and give appropriate feedback to pilots, according to our ambitious goal, providing them with reliable and rich human/machine interactions.

## Introduction

Increased operational capabilities of aircraft had considerably modified the missions of pilots and introduce new problematics. For example, long periods of intense and sustained cognitive activities induce cognitive fatigue that is known to impair the performance of reasoned cognitive processing tasks over a period and also to be one of the major risks of incidents/accidents in aviation [e.g., Holtzer et al. (2010), Marcus and Rosekind (2017), Dehais et al. (2018), and Dönmez and Uslu (2018)]. In this study, we aimed at furthering our understanding of the influence of cognitive fatigue on alarm detection in order to develop passive brain–computer interfaces (pBCIs) based on explainable artificial intelligence (AI). To achieve these ends and following previous studies (Dehais et al., 2018, 2019), we asked participants to perform an alarm-detection task during repeated landing sessions on a flight simulator. To accentuate the presence of cognitive fatigue, we also manipulated the mental workload. We tested whether a real glider flight in instruction prior to the experiment influences performance in the alarm detection task on a flight simulator. We hypothesized that (a) cognitive fatigue impairs alarm detection as a function of the mental workload, (b) cognitive fatigue modulates electrophysiological activities, and (c) these modulations can be used as a predictor of reduced pilot’s efficiency.

Previous studies have found that pilots’ performance is influenced by cognitive fatigue [e.g., Dehais et al. (2018, 2019), Keller et al. (2019), Rocha and Silva (2019), Quental et al. (2021), and Rosa et al. (2021)]. Implementing pBCI or neuro-adaptive technology is a relevant approach to study cognitive fatigue and to improve flight safety (Zander et al., 2016; Arico et al., 2017; Dehais et al., 2018). For example, Dehais et al. (2018) asked participants to perform four identical traffic patterns along with a secondary auditory task (i.e., oddball paradigm) in simulated and real flight conditions. The oddball paradigm is used as an indirect index of cognitive fatigue and alarm detection and allows evaluating the P300 component as well as the main frequency bands associated with cognitive fatigue. They found that pilots more erred when reporting the number of auditory probes during the second part of the experiment than during the first part. In other words, participants’ accuracy decreased with time on task. However, their small sample size did not allow them to statistically test the classification accuracies between the used features.

Empirically, previous findings showed that cognitive fatigue and mental workload have deleterious effects on stimulus-detection performance [e.g., Dehais et al. (2018, 2019)], whereas other findings showed an absence of a relationship between mental workload, cognitive fatigue, performance, and the occurrence of inattentional blindness [e.g., Bredemeier and Simons (2012), Beanland and Chan (2016), and Kreitz et al. (2016a,b)]. Unknown are the conditions under which cognitive fatigue or mental workload leads to poorer detection performance and their electrophysiological correlates. This is what we sought to know in this experiment.

The previously found attenuation of the P300 amplitudes reveals that inattentional deafness could result from an inability to automatically shift attention to the alarm that has been correctly detected or from an inability to process and recognize the warning (Giraudet et al., 2015b). However, we do not know whether event-related potentials (ERPs) and the time–frequency signal as a neural signature of inattentional deafness are good candidates as features to detect the occurrence of missed alarms.

The present experiment had two main goals. First, we investigated how alarm-detection changes associated with time on task interacted with other factors such as the cognitive workload or the type of previous activities (same task—flight instruction or different task—daily activities) and, *via* which mechanisms these factors influence performance. Second, we aimed at setting the scene to develop an EEG-based pBCI to detect alarm omissions to improve flight safety. Following previous studies on cognitive fatigue and alarm-detection tasks [e.g., Dehais et al. (2018)], participants had to perform an auditory task (i.e., oddball paradigm) during landing sessions. The mental workload was also manipulated to increase resulting cognitive fatigue. Based on previous findings that cognitive fatigue could impair performance by modulating attentional resources leaving fewer resources for tasks to perform [e.g., Chaudhuri and Behan (2004) and Holtzer et al. (2010)], two sets of hypotheses and predictions were tested in this study. The first hypothesis is that cognitive fatigue impairs alarm detection, resulting in increased alarm omissions in the fatigue group compared with the non-fatigue group and in the last landings compared with the first ones. The second hypothesis is that an efficient classification algorithm would be able to classify trials in which alarms were omitted and trials in which alarms were treated, based only on neurophysiological markers.

## Materials and Methods

### Participants

Twenty-four male students of the Ecole de l’Air et de l’Espace (EAE) [mean age: 22.6 (2.0) years; flight experience: 75.6 (79.6) h, including 44.7 (58.9) h of glider experience; Table 1] were recruited. Participants were divided into two groups of 12 each based on their activity preceding the experiment: (1) Instruction Flight Before the Experiment (IFBE) group and (2) No Flight Before the Experiment (NFBE) group.^1^ An informed consent was obtained from each participant prior to participation according to the Declaration of Helsinki.

**TABLE 1:** Participants’ characteristics.

Characteristics	NFBE group	IFBE group	*F*_(1,22)_
*N*	12	12	-
Mean age, in years (SDs, range)	23.2 (2.4, 21–27)	22.1 (1.6, 20–26)	1.75
Mean flight experience (glider and plane), in hours (SDs, range)	102.2 (97.3, 4–300)	49.1 (47.5, 4.5–150)	2.89
Mean flight experience (glider), in hours (SDs, range)	64.7 (59.3, 4–240)	24.7 (41.0, 4–150)	3.69

### Subjective Scales

At the beginning and end of the experimental session, participants rated their subjective level of fatigue (VASf; Lee et al., 1991), sleepiness (Karolinska’s Sleepiness Scale and VASs; Åkerstedt and Gillberg, 1990), and alertness (Samn-Perelli scale; Samn and Perelli, 1982).

### Tasks

#### Experimental Task in a Flight Simulator

The flight simulator of the EAE, used for training young student pilots, was used to conduct the experiment based on previous studies using flight simulators [e.g., Durantin et al. (2017) and Dehais et al. (2014, 2016, 2019)]. It simulates an ASK21 glider using the X-plane 11 software allowing a 135° view of the screen. No participant reported experiencing motion sickness or dizziness, nor had their visual perception been disturbed during the simulated flight.

#### Oddball Task

The auditory oddball task was coded and displayed using PsychoPy3 (Peirce, 2008). In this task, 100 pure tones, 1,000 or 1,100 Hz, at approximately 75 dB (20 dB above the ambient noise) were played, with 75% of standard sounds and 25% of target sounds. Participants had to respond to the auditory target (i.e., the alarm) by pressing a button on the joystick and ignore the frequent sounds. The frequency of the target sound was counterbalanced between participants. The intertrial interval was randomly set between 1.5 and 2.5 s to avoid anticipation and synchronization with brain rhythm (adapted from Dehais et al., 2019).

#### Flight Scenario

Participants performed six successive runs, in optimal weather conditions. Each run consisted of a normal approach and landing on the grass runway of the BA701 in Salon-de-Provence and lasted approximately 3–5 min. Each run was divided into two conditions of cognitive load, namely, a low cognitive load (LCL) condition (alarm detection task during the downwind leg) and a high cognitive load (HCL) condition (alarm detection task and backward counting task during the base leg, the final, and the landing). In the backward-counting task (Sweller, 2011), they had to mentally count backward in threes from 100 (e.g., 100-97-94…) and pronounce the result at the end of the landing.

### Procedure

The experience took place at the end of the afternoon. First, participants completed subjective questionnaires. Second, participants were trained for 5 min to handle the simulator and for 5 min to perform the oddball task. The experimental session lasted for approximately 1 h 30 min. At the end of the experiment, participants completed again the subjective questionnaires.

### Electroencephalogram Recording

The EEG apparatus contained 32 passive electrodes (R-Net-helmet, LiveAmp-Brain Products), positioned following the 10/20 international system, recording at a 1,000 Hz sampling rate. The offline preprocessing was achieved using the MATLAB EEGlab package (Iversen and Makeig, 2014). Data were first bandpass filtered between 1 and 40 Hz, the signal was re-referenced on the average of all electrodes, and an independent component analysis was performed to reject eye and muscle artifacts using the RUNICA function of EEG lab. The signal was then segmented into 1,200 ms epochs, starting 200 ms before the stimuli. The ERPs were computed using a baseline correction with the first 200 ms of each epoch. ERP amplitude was considered as the averaged amplitude over the time period, in each trial and then averaged for each participant. P300 was considered between 400 and 650 ms, and N100 was considered between 100 and 200 ms after the stimulus onset.^2^

The time-frequency analysis was achieved using the Brain Vision Analyzer 2 software (Brain Products, version 2.2.0.7383). Data were resampled at 512 Hz, and the power spectral density was extracted for δ (1–4 Hz), θ (4–8 Hz), α (8–12 Hz), and β (12–30 Hz) and then decomposed in low-β (12–16 Hz), mid-β (16–20 Hz), and high-β (20–30 Hz) bands for each trial (i.e., each epoch of 1.2 s). We focused our analyses on the Fz, Cz, Pz, and Oz electrodes.

The first three runs were considered as the beginning of the session while the last three runs were considered as the end of the session, in the subsequent analyses.

### Analyses

Based on the previous study (Dehais et al., 2019), we focused our EEG analyses on three electrodes for ERPs and on four electrodes for spectral power, in order to cut computation time from the perspective of real-time analyses. All statistical analyses were carried out using JASP software (JASP Team, 2020). *Post-hoc* tests were carried out with the Bonferroni’s correction for multiple comparisons, and a Greenhouse-Geisser correction was applied to respond to the sphericity condition when necessary.

## Results

### Subjective Fatigue Evaluation

No difference was observed between the beginning and the end of the experimental task (*Fs* < 1, *ps* > 0.5) for the Visual Analogous Scale of Fatigue, the Samn-Perelli scale, and the Karolinska scale.

### Experimental Task

#### Oddball Task

A 2 (group: NFBE and IFBE) × 2 (Time on task: beginning and end) × 2 (cognitive load: low and high) ANOVA with repeated measures and group as a between-subject factor was performed.

#### Detection Rate

**FIGURE 1:**
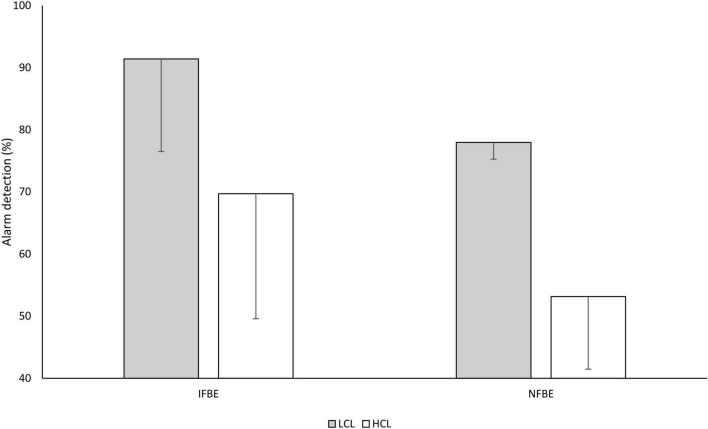
Mean detection rate in the oddball task across cognitive load conditions for the two groups of pilots. LCL corresponds to the low cognitive load condition, and HCL to the high cognitive load condition. Error bars represent the standard deviation of the mean.

The detection rate was higher in the LCL condition than in the HCL condition (83.8 vs. 61.2%), *F*_(1,14)_ = 102.92, *p* < 0.001, $\eta_{p}^{2}$ = 0.88, and participants in the IFBE group detected more alarms than the NFBE group (79.8 vs. 63.2%), *F*_(1,14)_ = 7.46, *p* = 0.016, $\eta_{p}^{2}$ = 0.35 (Figure 1). No other effect was found.

Participants responded faster in the LCL condition than in the HCL condition (547 vs. 609 ms), *F*_(1,13)_ = 22.66, *p* < 0.001, and $\eta_{p}^{2}$ = 0.64. No other effect was found on reaction times.

#### Electrophysiological Results

To compare electrophysiological signals between alarm detection and alarm omission, we focused our analyses on the HCL condition (participants missed more alarms in this condition). Data were analyzed with 2 (group: NFBE and IFBE) × 2 (time on task: beginning and end) × 3 (electrode: Fz, Cz, and Pz) × 2 (response: hit and miss) ANOVAs with repeated measures and group as the between-subject factor.

#### Event-Related Potentials

**FIGURE 2:**
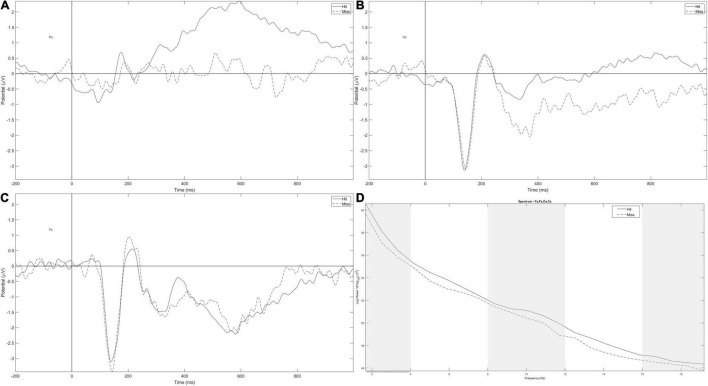
Event-related potential (ERP) measured on Pz **(A)**, Cz **(B)**, and Fz **(C)** for hit (full line) and miss trials (dotted line). **(D)** Averaged spectral power on Pz, Cz, Fz, and Oz for hits (full line) and missed trials (dotted line). Gray parts correspond to frequency bands of interest (delta, alpha, and mid-beta) and gray lines correspond to a significant difference between hit and miss trials in all conditions.

The P300 amplitude varied across electrodes, *F*_(2,32)_ = 13.45, *p* < 0.001, and η_*p*_^2^ = 0.46. The amplitude was larger on Pz than on Cz and Fz, respectively, *t* = −2.88, *p* = 0.02 and *t* = −5.17, *p* < 0.001 (Figures 2A–C). Numerically, the P300 amplitude measured on Pz is reduced in miss trials compared with hit trials, but this difference did not reach significance (Figure 2A).

The N100 amplitude also varied across electrodes, *F*_(2,32)_ = 8.57, *p* = 0.004, and $\eta_{p}^{2}$ = 0.35, being larger on Fz and Cz compared with Pz, respectively, *t* = −3.86, *p* = 0.001 and *t* = −3.198, *p* = 0.01 (Figures 2A–C).

#### δ, θ, α, and β Frequency Bands

The spectral power of the δ frequency band tended to be larger in hit trials (Figure 2D) compared with miss trials, *F*_(1,17)_ = 3.16, *p* = 0.093, and $\eta_{p}^{2}$ = 0.16. No other effect was found.

On the α frequency band, the significant effect of response (Figure 2D), *F*_(1,17)_ = 5.28, *p* = 0.035, and $\eta_{p}^{2}$ = 0.24, was qualified by the response × time on task interaction, *F*_(1,17)_ = 5.28, *p* = 0.035, and $\eta_{p}^{2}$ = 0.24. In the first three landings, the spectral power of the α frequency band was larger in hit trials compared with miss trials *t* = 3.248 and *p* = 0.016.

For the β frequency band, only the effect of the electrode was significant, *F*_(3,51)_ = 4.28, *p* = 0.053, and $\eta_{p}^{2}$ = 0.20, with a maximum on Oz compared with Fz and Cz, *t* = −2.89, *p* = 0.034 and *t* = 3.15, *p* = 0.017, respectively.

In the mid-β frequency band, *post-hoc* tests of the response × time on task × electrode × group interaction, *F*_(3,51)_ = 3.36, *p* = 0.075, and $\eta_{p}^{2}$ = 0.17, revealed that in the NFBE group, the spectral power was larger for hits than for miss trials at the beginning of the session, *t* = 4.74 and *p* = 0.003, and it was also larger in the beginning than at the end of the session, for hit trials, *t* = 4.06 and *p* = 0.048.

No effect was found on the θ frequency band.

### Single-Trial Classification

The classification pipeline was performed with the Scikit-Learn package of Python (Pedregosa et al., 2011). The first step of this process was to evaluate the performance of five classifiers [linear kernel, k-nearest neighbor (KNN), linear discriminant analysis (LDA), and random forest (RF) classifier] in participant-specific decoding of inattentional deafness, to distinguish trials in which the alarm was detected vs. trials in which alarms were omitted. Thus, classifiers were trained (80% of trials) and tested (20% of other trials) on individual pilots’ electrophysiological data, and features were tested according to previous results. Accuracy values of the different algorithms were analyzed with a five [classifier: linear support vector classification (SVC), KNN, SVC, LDA, and RF] × 7 (features: δ, α, mid-β, δ and α, δ and mid-β, α and mid-β, α and δ, and mid-β) ANOVA.

**FIGURE 3:**
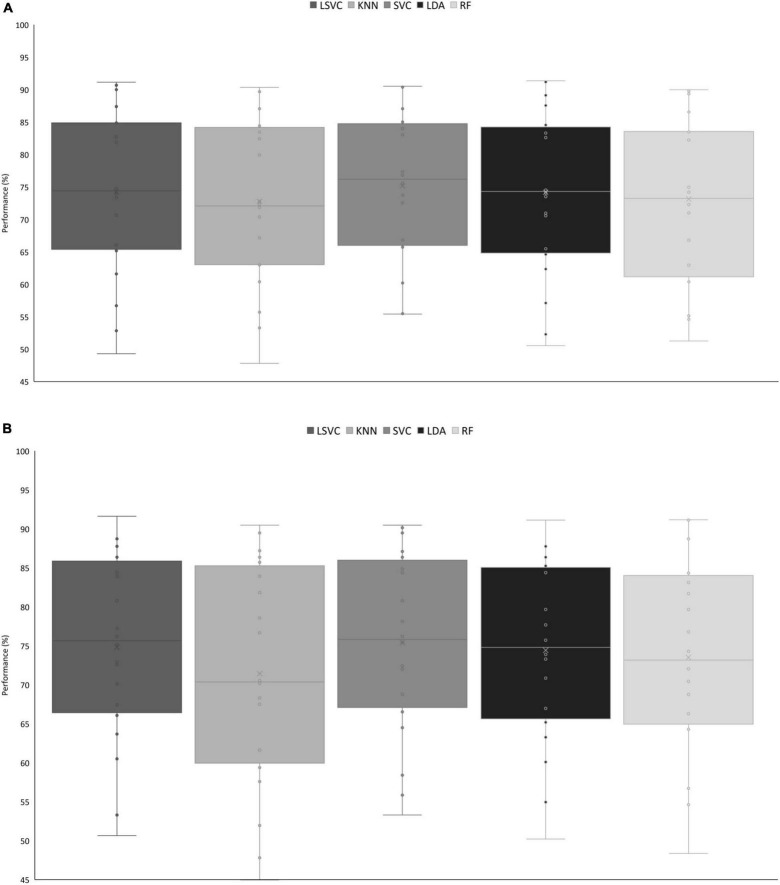
**(A)** Mean accuracy on the training dataset across classifiers. KNN < LSVC, *t* = 2.89, *p* = 0.050; KNN < SVC, *t* = −4.88, *p* < 0.001; RF < SVC, *t* = 4.12, *p* < 0.001. **(B)** Performance of classifiers on the test set. LSVC < KNN, *t* = 4.60, *p* < 0.001; KNN < SVC, *t* = −5.41, *p* < 0.001; KNN < LDA, *t* = −4.09, *p* < 0.001. KNN, k-nearest neighbor; SVC, support vector classification; RF, random forest.

The cross-validated scores obtained on the training set were first compared. The main effects of classifier, *F*_(4,76)_ = 7.48, *p* < 0.001, and $\eta_{p}^{2}$ = 0.28, and the interaction between classifier and features, *F*_(24,456)_ = 2.84, *p* < 0.001, and $\eta_{p}^{2}$ = 0.13, were significant. Across all features, the support vector machine (SVM) classifier reached the best performance of 75.2% on average (Figure 3A). For the SVM classifier, the most efficient configuration was the combination of the three frequency bands, with 75.9% of accuracy.

The inter-participant variability was quite high in the single-trial classification process, with accurate classification ranging from 47.1 to 90.5% across all configurations. However, generalization performance was then compared across configurations. In this analysis, the main effect of classifier was significant, *F*_(4,76)_ = 8.92, *p* < 0.001, and $\eta_{p}^{2}$ = 0.32. Across all features, the SVM classifier remained the most performant classifier on the testing dataset (Figure 3B).

The SVM algorithm aims at optimizing the classification accuracy and the distance between the boundary (which is a hyperplane) and each class. In fact, the algorithm is trained on the training dataset to minimize the expression of the form:


$$\left[\frac{1}{n}\,\sum_{i=1}^{n}\text{max}\,\left(0,1-y_{i}\,\left(w^{T}\,x_{i}-b\right)\right)\right]\,C\,{||w||}^{2}$$

where *n* is the number of data points, *w* is the normal vector to the hyperplane, *b* is the offset of the hyperplane from the origin, and *C*is the trade-off between correct classifications and distance separating the boundary hyperplane and each class.

For every classifier and feature, on average, the classifier performance exceeded the adjusted chance level of 61% based on Combrisson and Jerbi’s recommendations (Combrisson and Jerbi, 2015) to consider the number of available trials. We reached a maximum average performance of 76.4% (range: 57.7–90.5%) in participant-specific single-trial classification from the spectral power of δ and α frequency bands.

In a second step, data from all participants were taken altogether, and the different configurations were also tested for inter-participant classification. The main effect of classifier was significant, *F*_(4,16)_ = 40.67, *p* < 0.001, and $\eta_{p}^{2}$ = 0.91, showing that the KNN classifier is the least efficient classifier on the training set. We reached a maximum accuracy of 72.3% with the RF classifier and the combination of the three frequency bands.

## Discussion

This study aimed to implement an EEG-based pBCI with explainable AI to monitor alarm detections under cognitive fatigue in aviation. Cognitive fatigue could be accentuated by the previous activities (i.e., IFBE or NFBE). Participants had to perform flying sessions with a secondary auditory alarm detection task under HCL or LCL. Our results replicate previous findings on inattentional deafness (Dehais et al., 2014; Giraudet et al., 2015a,b; Causse et al., 2016) showing that participants performed better to detect alarms under LCL conditions compared with HCL conditions. However, the difference between the P300 evoked by detected alarms and the P300 evoked by omitted alarms did not reach significance. Also, we did not find the expected effect of cognitive fatigue on alarm detection performance, potentially because our task was not sufficiently difficult to induce high cognitive fatigue in such a short time. By comparing alarm detection with respect to alarm omission, we found increased α, δ, and β (only at the beginning of the session and for the NFBE group) power. Based on these three frequency bands, we performed a single-trial classification of alarm detection or omission. The SVM reached a mean of 76.4%, which is considered sufficient for pBCIs. In fact, there is a need to detect these attentional failures in cockpits, and as our classifier overpassed the adjusted chance level (i.e., 61%), this study showed that frequency features, and more specifically d and a bands, implemented in an SVC formed an efficient tool to assess auditory alarm misperception in simulated flight conditions, with a classification process adapted to each individual pilot. However, real-time implementation of pBCI is still difficult to achieve due to the large preprocessing step that is needed before classification. The challenge in these analyses was to reduce computation time and noise related to other factors (e.g., muscle activities). Possibly, neural oscillations are also related to movement and so, the differences we found between hits and miss trials could reflect not just inattentional deafness *per se* but also a difference in behavior. The same results have already been observed in previous studies using the same protocol and interpreted as inattentional deafness (Somon et al., 2022). As our goal was to classify alarm detection vs. alarm omission, motion-related variation could be used as an effective detection marker and be a true single-trial classification tool.

Another promising direction we investigated is to exploit explainable results from classification and machine learning computations. The objective is two-fold: to enlarge the experimentation process by relaying the result of the classification with an appropriate sequence of actions as a virtuous loop and ultimately to design new doctrines based on reliable and rich human/machine interactions. Such an understandable information (numerical, symbolic, and logical) constitutes a ground cognitive support and justifies the interpretability criterion (Lundberg and Lee, 2017) providing a good level of confidence at the operational level. The initial step is to look for explainable classification methods. For instance, a decision tree delivers logical rules characterizing the criteria separating alarm omission and alarm detection. The idea is to detect abnormal behaviors by our apparatus, and from sense-making information, to apply safely decision-making later (Bartheye and Chaudron, 2019, 2020), for instance, to enable a sequence of actions to be engaged, whether these actions are automatic or not. As a use-case, one can mention the situation in a cockpit characterized by a loss of attention of the pilot and his/her inability to continue his/her current mission. That is, the operator did not consciously detect the alarm although his brain processed the signal. It is, therefore, necessary to inform the operator that he has omitted the alarm (by feedback) and to adapt the work environment with the explainable AI to help him in his task so that he comes back in the loop.

The interpretability criterion provides a good level of confidence at the operational level and leads to the choice of the best candidate machine learning model, which will not necessarily be the most efficient in terms of classification, but one which would enable a sequence of actions to be engaged at the end, whether automatic or not. This choice of machine learning methods agreeing with the interpretability criterion is strongly restricted and one can mention decision trees and to a lesser extent RFs but there are great expectations to be associated with.

**FIGURE 4:**
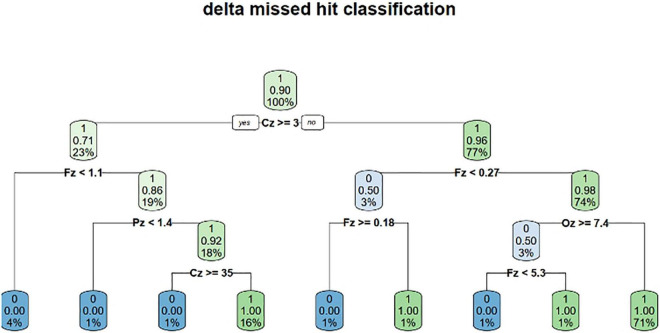
Decision tree generated by the decision tree algorithm.

To illustrate our discourse, we shall restrict the α frequency band full case study (2,855 individuals), and one can illustrate the principle on a single participant for sake of clarity (107 individuals) although the full case provides satisfactory results but obviously with more complicated formulas. The decision tree algorithm used is the Classification and Regression Trees (CART) algorithm (Breiman et al., 1984) and it provides the decision tree shown in Figure 4. The CART algorithm is a type of classification algorithm able to build a decision tree according to the Gini’s impurity index. This index computes the degree of probability of a specific variable that is wrongly being classified when chosen randomly. It works on categorical variables and provides outcomes either be “successful” or “failure” and hence conducts binary splitting only. The R statistical language implementation is called RPART (Recursive Partitioning And Regression Trees) (Therneau, 1997) and is available in a package of the same name. The control is defined according to an integer value, the minimum number of observations that must exist in a node for which the routine will even try to compute a split (4 for 107 individuals and 40 for 2855 individuals).

Starting from a normalized form of these decision rules, we generated the appropriate code in a static context or in dynamic context. In a static context, the missed hit logical rules generated in the R statistical language are the following:


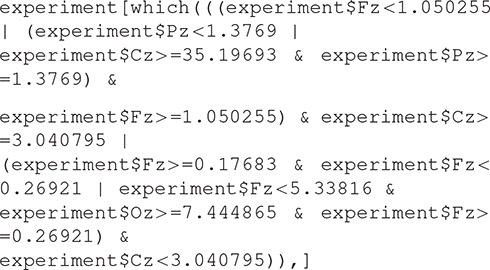


which means: print out all the columns of the table experiment whose lines correspond to missing hits as the column target shows (0 instead of 1) and the execution of this expression gives the classification result by extracting the right lines.


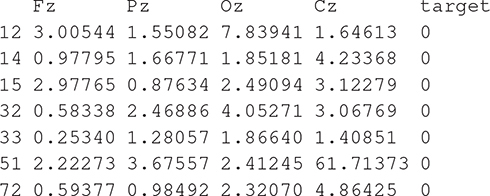


One can predict that way attention failure applying these rules regardless of the software involved (R, Python, Java, …). One can write a computer program as a case-based analysis by executing a task once a condition identifying a missing hit situation is true. If this situation characterizes a loss of attention for the pilot and his/her inability to continue his/her current mission, the associated task corresponds to crisis management. In a dynamic context, one can reengineer completely these rules according to a simulation platform intertwining actuators and sensors to be more creative on human/machine interactions. To summarize, our contribution to that field is to post-process the measurement and the acquisition mechanisms to deliver understandable statements able to be translated into program statements contributing to the global loop in studying cognitive fatigue.

## Data Availability Statement

The raw data supporting the conclusions of this article will be made available by the authors, without undue reservation.

## Ethics Statement

Ethical review and approval was not required for the study on human participants in accordance with the local legislation and institutional requirements. The patients/participants provided their written informed consent to participate in this study.

## Author Contributions

EM and LF conceived and planned the experiments and contributed to the interpretation of the results. EM carried out the experiments. LF supervised the project. All authors wrote the manuscript and approved the submitted version.

## Conflict of Interest

The authors declare that the research was conducted in the absence of any commercial or financial relationships that could be construed as a potential conflict of interest.

## Publisher’s Note

All claims expressed in this article are solely those of the authors and do not necessarily represent those of their affiliated organizations, or those of the publisher, the editors and the reviewers. Any product that may be evaluated in this article, or claim that may be made by its manufacturer, is not guaranteed or endorsed by the publisher.

## Footnotes

1 Participants in the NFBE group did not fly during the day of experiment but had daily activities such as classes or sports. They filled in a questionnaire with respect to these daily activities to inform experimenters whether they performed new and costful activities before the experiment, and this was not the case.

2 After evaluation of a control group doing the oddball task only.

## Abbreviations

pBCI: passive brain-computer interface
AI: artificial intelligence
ERP: event-related potential
IFBE: instruction flight before experiment
NFBE: no flight before experiment
VAS: visual analogous scale
LCL: low cognitive load
HCL: high cognitive load
SVC: support vector classification
KNN: K-nearest neighbors
LDA: linear discriminant analysis
RF: random forest.
